# Multi-Omics Revealed Breed Dominates over Plumage Color in Regulating Pigeon Meat Quality and Flavor

**DOI:** 10.3390/ani16071047

**Published:** 2026-03-30

**Authors:** Yuanxin Guan, Fei Ye, Xiaofei Xu, Jixiang Wei, Shen Liu, Miaomiao Yang, Jing Wang, Zhengsheng Li, Hai Xiang

**Affiliations:** 1 Guangdong Provincial Key Laboratory of Animal Molecular Design and Precise Breeding, School of Animal Science and Technology, Foshan University, Foshan 528225, China; 2 Zhongshan Shiqi Pigeon Breeding Co., Ltd., Zhongshan 528400, China; 3 Xinjiang Uygur Autonomous Region Academy of Animal Sciences, Urumqi 830011, China

**Keywords:** meat pigeon, meat quality, flavor substances, transcriptomics, metabolomics

## Abstract

Both breed and plumage color are considered potential genetic factors influencing meat quality in pigeons, yet their independent effects remain poorly distinguished. This study aimed to disentangle the regulatory roles of breed and plumage color on meat quality, nutritional composition, and flavor-related metabolites in meat pigeons. White-feathered (SQB) and grey-feathered (SQH) Shiqi pigeons were compared with European Mimas white pigeons (MMS) under identical rearing conditions. Slaughter performance, meat quality traits, and flavor profiles were assessed, followed by untargeted metabolomics and transcriptomics sequencing of pectoral muscle tissues. The results demonstrated that breed exerted a significant influence on carcass traits, water-holding capacity, collagen content, as well as the composition of fatty acids and free amino acids. In contrast, no notable disparity in meat quality was observed between the white- and gray-feathered varieties within the same Shiqi pigeon breed. A total of 114 and 205 differentially expressed metabolites (DEMs), and 11 and 327 differentially expressed genes (DEGs) were identified in plumage color and breed comparisons, respectively. Key flavor-associated metabolites, including glutathione, L-histidine, L-carnosine, and cytidine-5′-monophosphate, were identified as candidate biomarkers for breed-specific flavor differentiation. Breed is the dominant genetic factor determining meat quality and flavor in meat pigeons, while plumage color variation within breed has a limited impact. The identified pathways and regulatory networks provide actionable targets for the precision breeding and flavor enhancement of local pigeon breeds.

## Simple Summary

Meat quality and flavor are key factors influencing consumer preference for pigeon meat, but it remains unclear whether differences arise from breed or merely from feather color variants within a breed. By comparing white- and grey-feathered Shiqi pigeons with a European breed (Mimas white pigeon) using advanced molecular techniques, we found that breed overwhelmingly determines meat characteristics such as tenderness, fat content, and flavor compounds. No significant disparities were observed in meat quality indicators between the two feather color variants (white—feathered and gray—feathered) of the same Shiqu pigeon breed. Key molecules and metabolic pathways responsible for the superior flavor of Shiqi pigeon meat were identified. These findings provide breeders with precise targets to improve meat quality in local pigeon breeds, helping to meet market demand for high-quality products.

## 1. Introduction

Meat pigeons occupy an irreplaceable niche in China’s livestock sector due to their distinctive flavor, low fat content, and high protein profile [1]. As consumer demand for high-quality animal protein continues to rise, breeders have increasingly focused on genetic improvement to enhance both yield and meat quality. Among domestic breeds, the Chinese native Shiqi pigeon is renowned for its tender, juicy meat and unique clove-like aroma [2]. In contrast, the Mimas pigeon, a representative European meat breed, exhibits superior production performance and higher slaughter yields [3]. The slaughter weights of Shiqi pigeons and Mimas pigeons are approximately 510–600 g and 590–635 g, respectively, and both attain sexual maturity at around 180 days of age.

Recent advances in meat quality assessment have shifted from traditional sensory evaluation toward biomimetic sensing and high-throughput omics technologies. Electronic nose and electronic tongue systems enable quantitative flavor profiling by simulating human olfactory and gustatory mechanisms [4]. Metabolomics allows direct identification of flavor-related metabolites and elucidation of key regulatory pathways. For instance, by applying LC-MS-based lipidomics to compare metabolite and lipid profiles in different pig breeds, researchers revealed breed-specific muscle metabolic signatures [5]. Transcriptomics further complements these approaches by identifying differentially expressed genes (DEGs) that underlie meat quality traits. Integrated multi-omics analyses have proven powerful in deciphering the molecular networks governing muscle development and flavor formation. Yu et al. identified DEGs, differentially expressed proteins, and differential metabolites in porcine muscle, which were significantly enriched in Rap1 and Ras signaling pathways associated with meat quality [6]. At the genomic level, several studies have explored the genetic basis of meat quality traits in pigeons. Four novel SNP loci in exon 3 of the MyoD1 gene were identified and established their association with meat quality traits in domestic pigeons [7]. The ADSL gene was demonstrated to be closely correlated with carcass traits and meat quality in pigeons [8].

Despite the growing application of omics technologies in poultry meat research, the independent and interactive effects of breed and plumage color on meat flavor remain poorly distinguished. In particular, whether plumage color strains within the same breed exhibit divergent metabolic and transcriptional profiles affecting meat quality has not been systematically investigated. This knowledge gap hinders the precision breeding of local pigeon breeds tailored to specific flavor and quality standards. To date, no study has systematically disentangled the effects of breed versus plumage color on pigeon meat quality using integrated multi-omics approaches. This study addresses this gap by comparing white- and grey-feathered Shiqi pigeons with European Mimas pigeons. Through comprehensive phenotyping, targeted and untargeted metabolomics, transcriptomic sequencing, and integrative network analysis, this study aimed to quantify the relative contributions of breed and plumage color to meat quality and flavor and to identify key metabolic pathways and regulatory genes associated with flavor substance accumulation. The findings could provide a theoretical framework for the precision breeding and quality improvement of local meat pigeon breeds.

## 2. Materials and Methods

### 2.1. Ethics Statement

All experimental procedures were reviewed and approved by the Laboratory Animal Welfare and Animal Experimental Ethical Inspection Board of Foshan University (Approval ID: FOSU#20231018).

### 2.2. Sample Collection

Samples were collected from the Provincial Genetics Conservation Center for Shiqi Pigeon at Zhongshan Shiqi Pigeon Breeding Co., Ltd. (Zhongshan, China). Under identical feeding and management conditions, three pigeon groups—Shiqi white pigeons (SQB), Shiqi grey pigeons (SQH), and Mimas white pigeons (MMS)—were provided with ad libitum access to food and water. At 28 days of age, which is the typical marketing age for commercial meat pigeons, twelve squabs per group with similar body size and weight were randomly selected and slaughtered regardless of sex, as squab sales are not sex-restricted. Carcass weight (CW), half-eviscerated weight (HEW), eviscerated weight (EW), and left pectoral muscle weight (LPMW) were recorded, and the pectoral muscle percentage (PMP) was then calculated. Considering experimental feasibility and common practices in similar multi-omics studies on livestock and poultry [9,10,11], pectoral muscle samples from six birds per group (three males and three females) were selected for meat composition measurements and omics sequencing. To ensure sampling consistency, the same anatomical region was used for each type of analysis. Specifically, the right pectoralis major muscle was collected for meat quality analysis. The upper part of the left pectoralis major muscle adjacent to the wing base was utilized for transcriptomic and metabolomic determinations, whereas the lower part was used for electronic nose and electronic tongue tests. The remaining portion of the left pectoralis major muscle, together with the right pectoralis major muscle, was employed for other quality assessments and compositional analyses. Except for samples intended for routine meat quality tests, all other pectoral muscle samples were immediately excised, placed into cryovials containing liquid nitrogen, and snap-frozen for 5–10 min. Fully frozen samples were transferred to −80 °C ultra-low temperature freezers for storage. During transportation, samples were maintained on dry ice.

### 2.3. Determination of Meat Quality

Right pectoral muscle samples were stored at 4 °C for 24 h prior to pH measurement using a calibrated pH meter (Testo 205, Testo SE & Co., Lenzkirch, Germany). Samples were then heat-sealed in self-sealing bags and immersed in a 75 °C constant-temperature water bath for 30 min. After cooling, meat samples were trimmed to 2.5 cm × 1.0 cm dimensions. Shear force was measured using a digital muscle tenderness tester (C-LM3, Northeast Agricultural University, Harbin, China). Three replicate measurements were performed per sample, and the average value was used for statistical analysis. Texture profile analysis (TPA) was conducted using a texture analyzer (TA.XT Plus, Stable Micro Systems, Surrey, UK) equipped with a P75 probe. Parameters were set as follows: pre-test speed 1.00 mm/s, test speed 5.00 mm/s, post-test speed 5.00 mm/s, trigger force 6 g, and displacement 6.000 mm.

### 2.4. Determination of Meat Components

Moisture, salt, protein, and collagen contents, as well as lightness (L), redness (a*), and yellowness (b*) values, were determined using a rapid meat component analyzer (FoodScan™, FOSS, Hillerød, Denmark).

### 2.5. Determination of Meat Flavor

Odor profiles of right pectoral muscle were analyzed using an electronic nose (PEN3, AIRSENSE Analytics GmbH, Schwerin, Germany) equipped with 10 metal oxide semiconductor sensors. Taste profiles were analyzed using an SA402B electronic tongue (INSENT Inc., Atsugi, Japan) with five basic taste sensors and three aftertaste sensors. Measurements were performed according to previously described methods with minor modifications [12].

### 2.6. Targeted Metabolomics Analysis

Short-chain and medium-to-long-chain fatty acids in left pectoral muscle were quantified using a Thermo Scientific Trace 1310 GC system coupled with an ISQ LT mass spectrometer (Thermo Fisher Scientific, Waltham, MA, USA) under specified temperature and flow rate conditions, as described by Wang et al. [13]. Free amino acids were analyzed using an Agilent 1290 Infinity UHPLC system coupled with a 6500+ QTRAP^®^ mass spectrometer (SCIEX, Framingham, MA, USA), following the protocol described by Jeong et al. [14].

### 2.7. Untargeted Metabolomics Analysis

Metabolite profiling was performed using LC-MS/MS on an Agilent 1290 Infinity LC system coupled with an AB SCIEX Triple TOF 6600 mass spectrometer (SCIEX, Framingham, MA, USA), according to Shi et al. [15]. Metabolite screening, principal component analysis (PCA), and orthogonal partial least squares discriminant analysis (OPLS-DA) were conducted using the MetaboAnalystR package (version 1.0, 2018) [16]. Differential metabolites (DEMs) were identified based on fold change (FC) > 1.5 and *p* < 0.05 (VIP > 1 criterion was removed due to unsatisfactory model fitting). Pathway enrichment analysis of DEMs was performed using the KEGG database.

### 2.8. Transcriptome Analysis

Total RNA was extracted from left pectoralis muscle using TRIzol reagent (Invitrogen, Carlsbad, CA, USA). Qualified samples were used to construct sequencing libraries with the Illumina NEBNext^®^ Ultra™ RNA Library Prep Kit (Illumina, San Diego, CA, USA) and then PE150 sequenced on an Illumina NovaSeq 6000 platform. After quality control and sequence alignment, differential gene expression analysis was carried out using the DESeq2 R package algorithm [17]. In accordance with prior research [18], genes that satisfied the criteria of |log_2_FC| > 1.5 and an adjusted *p*-value (*p*-adj) < 0.05 were delineated as DEGs. Gene Ontology (GO) and KEGG pathway enrichment analyses were conducted using the DAVID online platform with a significance threshold of *p* < 0.05.

### 2.9. Statistical Analysis

Phenotypic data were analyzed using SPSS 27 (IBM, Armonk, NY, USA) and expressed as mean ± standard error (SE). Normality and homogeneity of variances were examined prior to analysis. Independent-samples *t*-tests were employed to evaluate inter-group differences. Statistical significance was set at *p* < 0.05.

## 3. Results

### 3.1. Carcass Traits Comparison

**Figure 1:**
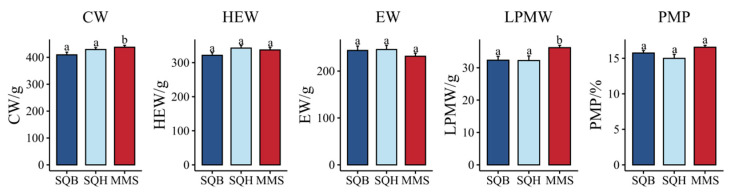
Carcass traits of meat pigeons with different plumage colors and breeds. CW: carcass weight; HEW: half-eviscerated weight; EW: eviscerated weight; LPMW: left pectoral muscle weight; PMP: pectoral muscle percent. Different letters indicate significant differences (*p* < 0.05). SQB: Shiqi white pigeon; SQH: Shiqi grey pigeon; MMS: Mimas white pigeon.

Carcass traits were compared between SQB and SQH (plumage color groups) and between SQB and MMS (breed groups). No significant differences in slaughter performance were observed between the two plumage color groups (*p* > 0.05). However, in the breed comparison, carcass weight (*p* = 0.008) and left pectoral muscle weight (*p* = 0.003) were significantly lower in SQB than in MMS, while other carcass parameters showed no significant differences (Figure 1). The findings demonstrate that feather color exhibits negligible effects on carcass characteristics among Shiqi pigeons of the same breed. Breed constitutes the principal determinant of key slaughter performance indices.

### 3.2. Meat Quality, Texture Profile and Composition Comparison

**Figure 2:**
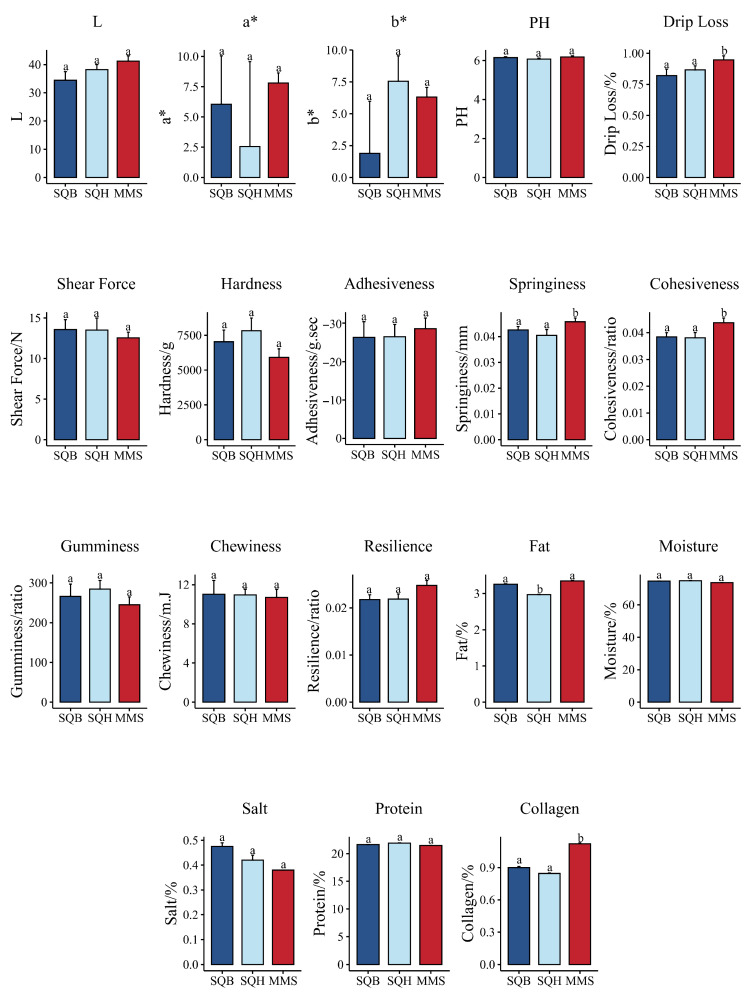
Meat quality, texture profile and composition of meat pigeons with different plumage colors and breeds. Different letters indicate significant differences (*p* < 0.05). SQB: Shiqi white pigeon; SQH: Shiqi grey pigeon; MMS: Mimas white pigeon.

Meat quality analysis revealed no significant differences in both meat quality and texture profile between plumage color groups for any parameter (*p* > 0.05) (Figure 2). In contrast, drip loss was significantly lower in SQB than in MMS (*p* = 0.004), indicating superior water-holding capacity. Moreover, springiness (*p* = 0.012) and cohesiveness (*p* = 0.009) were significantly lower in SQB than in MMS, suggesting breed-specific textural characteristics. No significant differences were observed for color parameters, pH, shear force, hardness, adhesiveness, gumminess, chewiness, or resilience (*p* > 0.05) (Figure 2). Regarding meat composition, fat content was significantly higher in SQB than in SQH (*p* = 0.011) in plumage color comparisons. In breed comparisons, collagen content was significantly lower in SQB than in MMS (*p* = 0.003) (Figure 2). No significant differences were observed for salt, moisture, or protein content between SQB and MMS.

### 3.3. Meat Flavor Profiles Comparison

**Figure 3:**
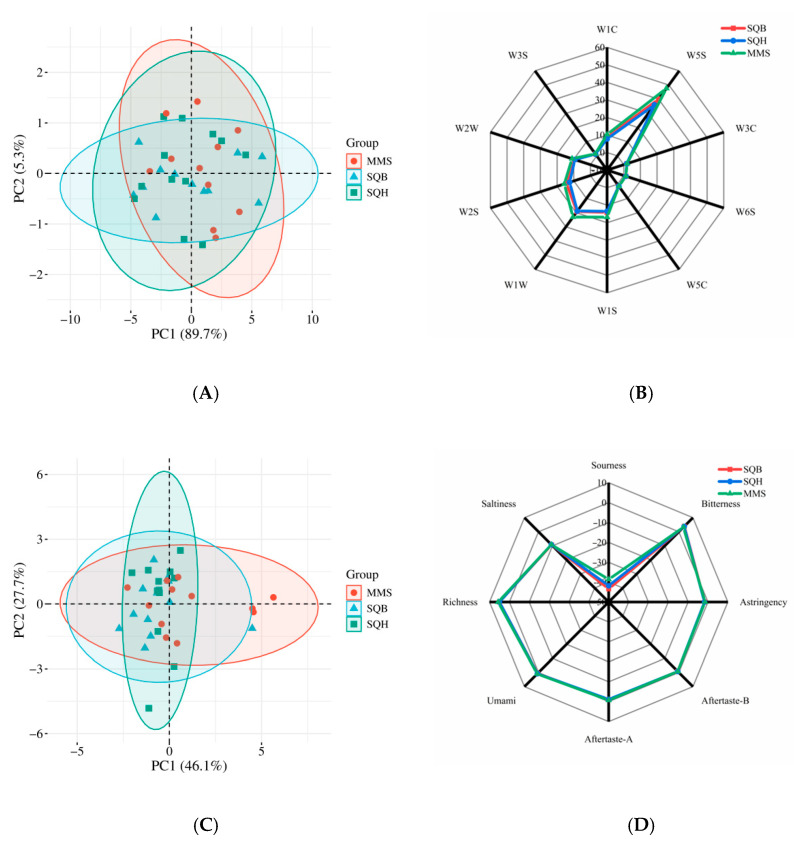
PCA and radar analyses of pectoral muscle odor and taste in three pigeon groups. (**A**) PCA plot of pectoral muscle odor; (**B**) radar chart of pectoral muscle odor; (**C**) PCA plot of pectoral muscle taste; (**D**) radar chart of pectoral muscle taste. SQB: Shiqi white pigeon; SQH: Shiqi grey pigeon; MMS: Mimas white pigeon.

Electronic nose analysis revealed that the cumulative contribution rate of principal components reached 95.0% (PC1: 89.7%, PC2: 5.3%), fully characterizing the odor profiles of the three groups. However, samples from different groups were mixed and scattered (Figure 3A), showing no obvious clustering based on breed or plumage color. The radar chart also showed no substantial differences in pectoral muscle odor among groups, although six sensors (W1W, W1S, W2S, W5S) exhibited relatively higher response values in MMS than in SQB and SQH (Figure 3B), corresponding to sensitivity toward inorganic sulfides, short-chain hydrocarbons, alcohols/aldehydes/ethers, and nitrogen oxides, respectively. Similarly, PCA of electronic tongue data showed similar patterns among groups (Figure 3C). All five basic taste attributes (sourness, bitterness, astringency, saltiness, umami) and three aftertaste attributes (aftertaste-A, aftertaste-B, richness) were detected, with no marked differences among groups (Figure 3D).

### 3.4. Fatty Acid and Free Amino Acid Profiles

In plumage color comparisons (Table 1), five of seven short-chain fatty acids (acetic acid, butyric acid, caproic acid, isobutyric acid, and valeric acid) and two medium-to-long-chain fatty acids (C23:0 and C24:0) exhibited significantly lower levels in SQB than in SQH (*p* < 0.05). However, total EFA, SFA, UFA, MUFA, PUFA, ω-6 PUFA, and ω-3 PUFA contents did not differ significantly between these two groups. In breed comparisons (Table 1), SQB showed significantly lower levels of isobutyric acid, C10:0, C17:0, C17:1T, C18:2N6, C18:3N3, C19:1N12T, C19:1N9T, C20:5N3, and C22:5N3 than MMS (*p* < 0.05). In contrast, C22:1N9T, C22:4, and C24:1 were significantly higher in SQB (*p* < 0.05). Total EFA, PUFA, ω-6 PUFA, and ω-3 PUFA contents were significantly lower in SQB than in MMS (*p* < 0.05). These results demonstrate that breed, but not plumage color, significantly influences overall fatty acid composition.

For free amino acids (Table 2), valine content was significantly higher in SQB than in SQH (*p* = 0.008). However, total SAA, DAA, BCAA, and EAA contents, as well as their ratios to total amino acids, did not differ significantly between plumage color groups. In breed comparisons (Table 2), four free amino acids (methionine, phenylalanine, tyrosine, and valine) were significantly higher in SQB than in MMS (*p* < 0.05), while cystine and proline were significantly lower in SQB (*p* < 0.05). In addition, total SAA, DAA, BCAA, and EAA contents, as well as the DAA/TAA and EAA/TAA ratios, were significantly higher in SQB than in MMS (*p* < 0.05). These findings confirm that breed is the predominant factor shaping free amino acid profiles.

### 3.5. Distinct Metabolic Signatures of Breed and Plumage Color

**Figure 4:**
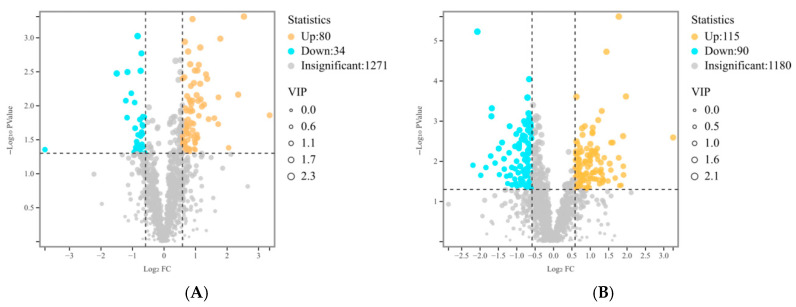

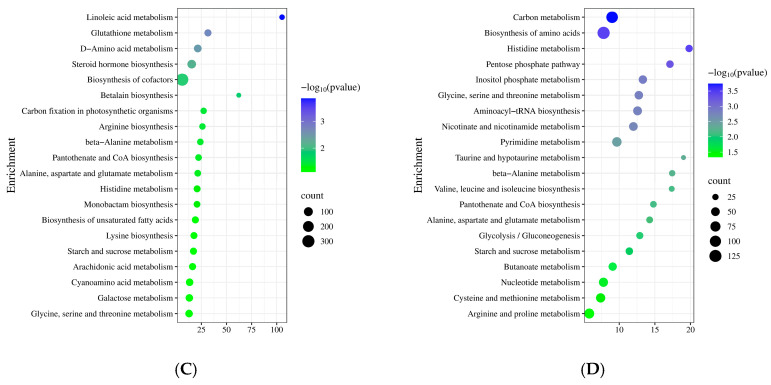
Pectoral muscle metabolic profile of different pigeon groups. (**A**) Volcano plot of DEMs in plumage color comparison (SQB vs. SQH); (**B**) Volcano plot of DEMs in breed comparison (SQB vs. MMS); (**C**) KEGG enrichment bubble chart of DEMs in plumage color comparison; (**D**) KEGG enrichment bubble chart of DEMs in breed comparison. SQB: Shiqi white pigeon; SQH: Shiqi grey pigeon; MMS: Mimas white pigeon. (Notes: The vertical dashed line indicates the threshold of fold change, while the horizontal dashed line indicates the threshold of statistical significance.)

**Figure A1:**
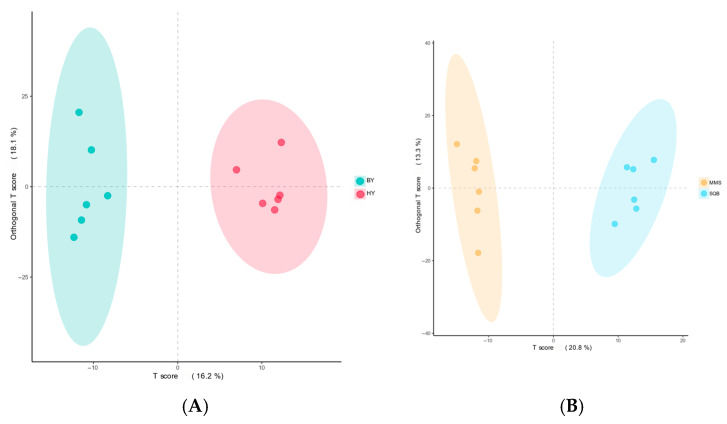
PCA plot of odor-related metabolites. (**A**) PCA plot of odor-related metabolites in plumage color comparison (SQB vs. SQH); (**B**) PCA plot of odor-related metabolites in breed comparison (SQB vs. MMS).

**Figure A2:**
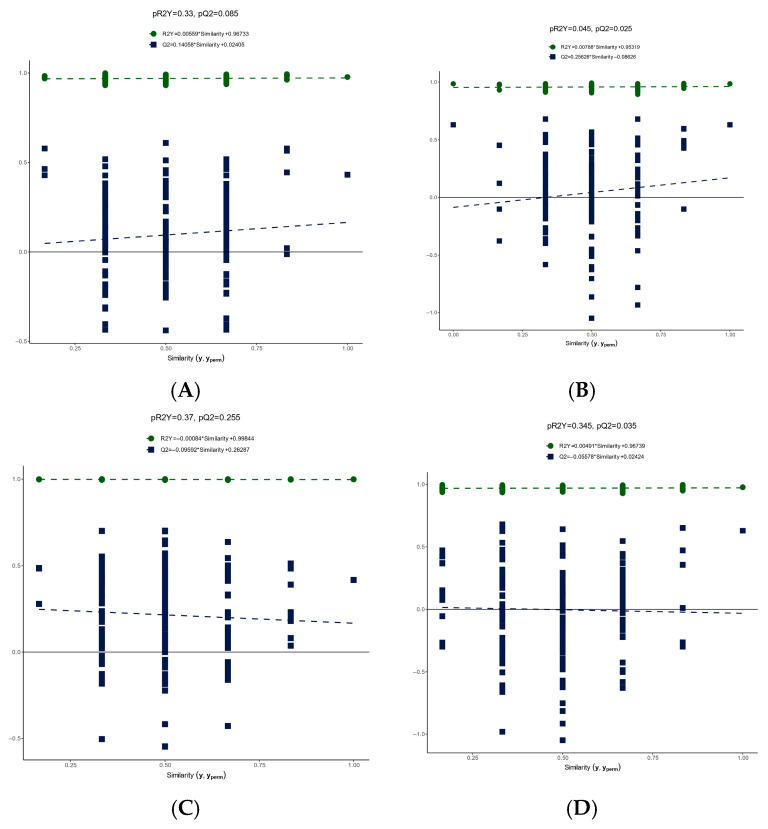
OPLS-DA permutation test. (**A**) Positive ion mode: OPLS-DA permutation test plot in plumage color comparison (SQB vs. SQH); (**B**) Negative ion mode: OPLS-DA permutation test plot in plumage color comparison (SQB vs. SQH); (**C**) Positive ion mode: OPLS-DA permutation test plot in breed comparison (SQB vs. MMS); (**D**) Negative ion mode: OPLS-DA permutation test plot in breed comparison (SQB vs. MMS). The obtained parameters are as follows: For breed comparison in positive ion mode: R^2^ = 0.33, Q^2^ = 0.085; For breed comparison in negative ion mode: R^2^ = 0.37, Q^2^ = 0.255; For plumage color comparison in positive ion mode: R^2^ = 0.045, Q^2^ = 0.025; For plumage color comparison in negative ion mode: R^2^ = 0.345, Q^2^ = 0.035.

Principal component analysis (PCA) demonstrated that samples from the same group exhibited closely clustered distributions with similar patterns and were clearly distinguishable from those of other groups (Figure A1). Along with the results of OPLS-DA (Figure A2), these indicate that the sample selection per group is reliable and meets the requirements of this exploratory study for the preliminary characterization of metabolic profiles across different pigeon breeds and populations. A total of 114 DEMs were identified between the plumage color groups, including 34 down-regulated and 80 up-regulated in SQH vs. SQB (Figure 4A). In contrast, 205 DEMs were found between breed groups, with 90 down-regulated and 115 up-regulated in MMS vs. SQB (Figure 4B). DEMs in plumage color groups were significantly enriched in 13 pathways, including linoleic acid metabolism, glutathione metabolism, D-amino acid metabolism, steroid hormone biosynthesis, histidine metabolism, arginine biosynthesis, and alanine/aspartate/glutamate metabolism (Figure 4C). In breed groups, DEMs were enriched in 38 pathways, among which carbon metabolism, amino acid biosynthesis, histidine metabolism, alanine/aspartate/glutamate metabolism, pyrimidine metabolism, and beta-alanine metabolism were directly linked to muscle development and flavor regulation (Figure 4D).

### 3.6. Transcriptional Landscapes Comparison

**Figure 5:**
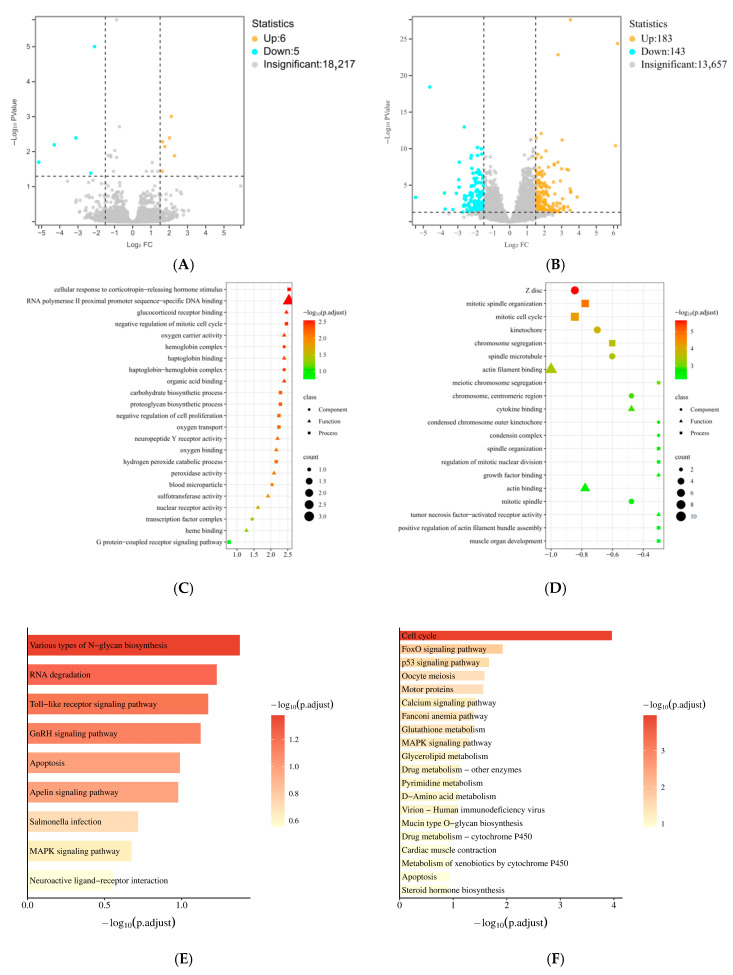
Pectoral muscle transcriptomic profile of different pigeon groups. (**A**) Volcano plot of DEGs in plumage color comparison (SQB vs. SQH); (**B**) Volcano plot of DEGs in breed comparison (SQB vs. MMS); (**C**) GO enrichment of DEGs in plumage color comparison; (**D**) GO enrichment of DEGs in breed comparison; (**E**) KEGG enrichment of DEGs in plumage color comparison; (**F**) KEGG enrichment of DEGs in breed comparison. SQB: Shiqi white pigeon; SQH: Shiqi grey pigeon; MMS: Mimas white pigeon. (Notes: The vertical dashed line indicates the threshold of fold change, while the horizontal dashed line indicates the threshold of statistical significance).

A total of 11 DEGs were identified in plumage color comparison, including 6 up-regulated and 5 down-regulated in SQH vs. SQB (Figure 5A). Meanwhile, 327 DEGs were found between breed groups, with 184 down-regulated and 143 up-regulated in MMS vs. SQB (Figure 5B). GO enrichment analysis showed that DEGs in plumage color groups were enriched in 21 significant GO terms (7 BP, 10 MF, 4 CC), while DEGs in breed groups were enriched in 50 significant GO terms (27 BP, 11 MF, 12 CC). The most significant GO terms in plumage color groups included glucocorticoid receptor binding, organic acid binding, and carbohydrate biosynthetic process (Figure 5C). Meanwhile, the most significant GO terms in breed groups included mitotic spindle organization, growth factor binding, mitotic cell cycle, chromosome segregation, and cytokine binding (Figure 5D). KEGG analysis revealed that DEGs in plumage color groups were significantly enriched in only one pathway, various types of N-glycan biosynthesis (Figure 5E); whereas DEGs in breed groups were significantly enriched in eight pathways, including cell cycle, FOXO signaling pathway, p53 signaling pathway, calcium signaling pathway, glutathione metabolism, and glycerolipid metabolism (Figure 5F), all closely associated with muscle development and meat flavor.

## 4. Discussion

This research represents a preliminary, exploratory, multi-omics characterization investigation. Our results preliminarily indicate that breed is the dominant genetic determinant, while plumage color variation within breed exerts minimal influence on carcass traits, meat quality parameters, nutritional composition, and flavor-related metabolite profiles. This hierarchical understanding of genetic factors is critical for designing precision breeding strategies in meat pigeons.

In terms of carcass traits, comparative analysis of slaughter performance across different meat pigeon breeds showed that the carcass weight and breast muscle weight of SQB were generally lower than those of MMS. Nevertheless, there was no significant difference in breast muscle percentage between the two breeds. SQB exhibited superior water-holding capacity, as reflected by significantly lower drip loss compared to MMS, along with higher intramuscular fat content. These traits are well-established contributors to brighter meat color and improved tenderness [19]. The significantly lower collagen content in SQB relative to MMS further supports its tenderness advantage, since collagen cross-linking density is positively correlated with shear force and hardness [20,21]. Notably, all three groups displayed pH values within the optimal range (5.97–6.4) characteristic of high-quality pigeon meat [22], confirming their status as premium meat-type breeds. Collectively, these phenotypic differences underscore that breed-specific genetic backgrounds dictate fundamental muscle biochemical properties that translate into distinct eating qualities.

Regarding flavor and nutritional value, the UFA/SFA ratios of all three breeds ranged from 2.02 to 2.27, substantially exceeding the nutritional quality benchmark, thereby affirming their high nutritional value. SFAs from meat have long been regarded as risk factors for cardiovascular diseases. The SQB breed had a significantly lower SFA content than MMS, implying a potential advantage of Shiqi pigeon in reducing the risk of cardiovascular diseases. UFAs are among the essential nutrients that must be obtained through dietary intake. Notably, the UFA content in pigeon meat is far higher than that in pork, which also explains why SQB has become a meat product of growing interest and preference among consumers. Studies have found that a higher UFA content in animal tissues tends to facilitate the thermal oxidation of fats during cooking, thereby generating distinct aromatic compounds [23]. The predominance of aspartic acid among free amino acids and the high proportion of umami amino acids (43–48% of total amino acids) underpin the characteristic savory flavor of pigeon meat [24,25]. Moreover, the significantly higher EAA/TAA ratio in SQB compared to MMS indicates superior protein nutritional quality [26], reinforcing the breed-specific nutritional hierarchy. While plumage color differences led to isolated changes in individual fatty acids or amino acids, no consistent alteration in the overall composition of fatty acid classes or total amino acid pools was observed. Thus, breed acts as the primary determinant of both the sensory and nutritional dimensions of pigeon meat.

To elucidate the molecular basis of these phenotypic divergences, we performed metabolomic and transcriptomic profiling. KEGG enrichment of DEMs identified glutathione metabolism as the principal pathway distinguishing plumage color strains, whereas histidine metabolism, beta-alanine metabolism, and phenylalanine/tyrosine/tryptophan biosynthesis were strongly breed-discriminatory. These pathways have been independently implicated in meat flavor regulation in chickens [27] and sheep [28], suggesting evolutionary conservation of core flavor-associated metabolic modules across avian and mammalian species. Moreover, the activation of phenylalanine, tyrosine, and tryptophan biosynthesis during metabolism can significantly increase the contents of phenylalanine and tyrosine. Several DEMs enriched in these pathways—glutathione, γ-glutamylcysteine, L-histidine, carnosine, histamine, L-phenylalanine, and DL-tyrosine—are known flavor modulators. As precursor substances for volatile flavor components in pigeon meat [29], these amino acids provide a sufficient material basis for the formation of characteristic flavor in pigeon meat products. Glutathione synergistically enhances umami perception [30]; L-histidine amplifies saltiness and stabilizes muscle protein structure [31]; carnosine promotes pyrazine formation during heating, generating roasted aroma notes [32].

Although 114 differential metabolites were identified between plumage color groups, only a few of them (e.g., aspartic acid and glutathione) are related to flavor. The majority of the remaining differential metabolites (e.g., salannin, anisomycin, brucine) are predominantly exogenous substances or metabolites unrelated to meat flavor. For example, 5,6-dihydroxyindole-2-carboxylic acid is involved in feather pigment synthesis and deposition [33], whereas gamma-glutamylcysteine mainly serves as an antioxidant and maintains cellular homeostasis [34]. These compounds do not directly contribute to core meat quality traits such as flavor, tenderness, umami, or aroma. This further indicates that plumage-related differential metabolites are rarely involved in the regulation of core meat flavor characteristics. The differential accumulation of these metabolites between SQB and MMS therefore provides a plausible metabolic explanation for the preferred flavor profile of Shiqi pigeon meat.

Transcriptome analysis revealed that breed-specific DEGs were significantly enriched in pathways governing cytoskeletal organization, cell cycle regulation, calcium signaling, and p53 signaling. The degradation rate of myofibrillar proteins, which is cytoskeleton-dependent, directly influences tenderness and juiciness [35]. Calcium signaling mediates tenderness development through downstream calpain-catalyzed proteolysis [36], explaining the divergent tenderness phenotypes observed between SQB and MMS. Genes correlated with the p53 signaling pathway may indirectly regulate fatty acid oxidation and intramuscular fat deposition through modulation of OXR1 expression [37], thereby linking transcriptional regulation to the accumulation of flavor precursors. Notably, only 11 DEGs were detected between plumage color strains, and these genes were not enriched in any pathway directly related to muscle metabolism or flavor, further corroborating the phenotypic evidence that feather color variants within the same breed have negligible impact on meat quality.

Our findings align with recent reports in chickens [9,38,39] and other farm animals [40,41], where breed exerted predominant effects on muscle metabolite profiles while plumage color variants showed minimal divergence. This convergence across poultry species suggests that intensive selection for production traits may overshadow pigmentation-related metabolic divergence. However, the central role of histidine metabolism and the specific gene-metabolite associations identified here appear to be pigeon-specific, warranting further comparative investigations across domesticated birds. Taken together, this multi-omics investigation establishes that breed, rather than plumage color, is the predominant genetic driver of meat quality and flavor in meat pigeons. The identified pathways and gene-metabolite networks offer actionable targets for marker-assisted selection and for developing molecular tools to accelerate the genetic improvement of local pigeon breeds. Moreover, our study provides a paradigmatic framework for disentangling confounded genetic factors in other livestock species where multiple traits are under simultaneous selection.

A limitation of this study is that DEGs were not verified using qRT-PCR. As an exploratory investigation, the primary emphasis of this research was to compare disparities in meat quality and gene expression among diverse breeds and feather colors through multi-omics analysis, rather than on the functional validation of individual key genes. However, such validation is of great significance for confirming the reliability of gene expression profiles. In future research, validate key meat-quality-related DEGs by means of qRT-PCR are expected to included to explore their expression patterns and regulatory mechanisms.

## 5. Conclusions

The plumage color of Shiqi pigeons within the same breed does not exert a significant influence on meat quality, nutritional composition, or sensory flavor attributes. Rather, breed serves as the primary determinant of these characteristics. Shiqi white pigeons (SQB) exhibit superior meat quality and flavor characteristics compared to Mimas white pigeons (MMS). Metabolomic and transcriptomic profiling revealed 114 and 205 DEMs, and 11 and 327 DEGs in plumage color and breed comparisons, respectively, with breed effects overwhelmingly predominant. Key flavor-associated metabolites—including glutathione, L-histidine, L-carnosine, and cytidine-5′-monophosphate—were identified as candidate biomarkers for breed-specific flavor differentiation. These findings provide a molecular framework for precision breeding programs targeting improved meat quality and flavor in local meat pigeon breeds and establish a foundation for comparative meat science across poultry species.

## Figures and Tables

**Table 1:** Fatty acid profiles of different pigeon groups.

Item	SQB (*n* = 6)	SQH (*n* = 6)	MMS (*n* = 6)
Acetic acid	166.28 ± 110.65 ^a^	361.32 ± 172.81 ^b^	213.17 ± 115.73 ^a^
Butyric acid	9.73 ± 5.03 ^a^	27.68 ± 12.88 ^b^	15.52 ± 6.03 ^a^
Caproic acid	1.58 ± 0.62 ^a^	4.51 ± 2.14 ^b^	2.61 ± 0.85 ^a^
Isobutyric acid	0.52 ± 0.30 ^a^	0.90 ± 0.16 ^b^	0.57 ± 0.35 ^b^
Isovaleric acid	0.07 ± 0.12	0.11 ± 0.10	0.00 ± 0.00
Propionic acid	0.96 ± 0.17	1.12 ± 2.24	0.75 ± 0.42
Valeric acid	0.41 ± 0.10 ^a^	0.67 ± 0.20 ^b^	0.54 ± 0.21 ^a^
C10:0	0.15 ± 0.03 ^a^	0.17 ± 0.04 ^a^	0.18 ± 0.02 ^b^
C12:0	0.3 ± 0.05	0.29 ± 0.04	0.29 ± 0.03
C13:0	0.08 ± 0.02	0.09 ± 0.01	0.08 ± 0.02
C14:0	3.32 ± 0.89	2.79 ± 0.43	3.18 ± 0.47
C14:1	0.91 ± 0.20	0.76 ± 0.15	0.75 ± 0.28
C14:1T	0.12 ± 0.07	0.14 ± 0.06	0.07 ± 0.05
C15:0	0.62 ± 0.11	0.6 ± 0.11	0.67 ± 0.08
C15:1	0.17 ± 0.03	0.19 ± 0.03	0.16 ± 0.05
C15:1T	0.10 ± 0.04	0.12 ± 0.03	0.10 ± 0.04
C16:0	279.54 ± 51.59	300.12 ± 43.38	322.65 ± 57.72
C16:1	79.52 ± 16.65	78.76 ± 7.27	71.66 ± 19.42
C16:1T	0.49 ± 0.06	0.5 ± 0.02	0.51 ± 0.11
C17:0	1.86 ± 0.37 ^a^	1.92 ± 0.48 ^a^	2.53 ± 0.52 ^b^
C17:1	4.08 ± 0.93	4.97 ± 0.70	4.26 ± 0.85
C17:1T	0.58 ± 0.09 ^a^	0.58 ± 0.08 ^a^	0.74 ± 0.08 ^b^
C18:0	222.08 ± 41.44	224.46 ± 28.73	240.68 ± 31.02
C18:1N12	14.97 ± 2.37	15.61 ± 4.76	15.39 ± 3.89
C18:1N12T	1.08 ± 0.16	1.2 ± 0.29	0.99 ± 0.13
C18:1N7	62.6 ± 15.04	61.17 ± 5.07	65.51 ± 10.49
C18:1N7T	0.42 ± 0.03 ^a^	0.46 ± 0.05 ^a^	0.56 ± 0.04 ^b^
C18:1N9C	455.33 ± 119.48	455.87 ± 56.63	516.7 ± 137.14
C18:1N9T	2.16 ± 0.31	2.16 ± 0.13	2.07 ± 0.21
C18:2N6	329.71 ± 75.35 ^a^	308.94 ± 56.16 ^a^	469.21 ± 82.54 ^b^
C18:2N6T	0.3 ± 0.07	0.49 ± 0.25	0.4 ± 0.28
C18:3N3	2.93 ± 0.69 ^a^	2.39 ± 0.44 ^a^	5.12 ± 0.80 ^b^
C18:3N6	1.08 ± 0.11	1.09 ± 0.11	1.02 ± 0.08
C19:1N12T	0.91 ± 0.19 ^a^	0.95 ± 0.16 ^a^	1.25 ± 0.20 ^b^
C19:1N9T	0.63 ± 0.11 ^a^	0.68 ± 0.08 ^a^	0.87 ± 0.11 ^b^
C20:0	1.65 ± 0.35	1.78 ± 0.18	1.71 ± 0.23
C20:1	4.62 ± 1.43	4.84 ± 1.21	3.86 ± 0.73
C20:1T	0.15 ± 0.06	0.16 ± 0.03	0.14 ± 0.02
C20:2	3.4 ± 0.97	3.13 ± 0.08	3.15 ± 0.43
C20:3N3	0.34 ± 0.06	0.33 ± 0.04	0.35 ± 0.04
C20:3N6	6.61 ± 1.12	7.82 ± 1.21	6.91 ± 0.66
C20:4N6	83.08 ± 13.70	90.45 ± 18.5	88.94 ± 11.49
C20:5N3	2.41 ± 0.51 ^a^	2.63 ± 0.49 ^a^	3.33 ± 0.74 ^b^
C21:0	0.15 ± 0.02	0.17 ± 0.02	0.15 ± 0.02
C22:0	0.39 ± 0.08	0.48 ± 0.13	0.41 ± 0.09
C22:1N9	1.47 ± 0.59	1.56 ± 0.31	1.02 ± 0.11
C22:1N9T	0.13 ± 0.06 ^a^	0.11 ± 0.03 ^a^	0.06 ± 0.01 ^b^
C22:2	0.21 ± 0.10	0.21 ± 0.05	0.16 ± 0.03
C22:4	15.82 ± 2.4 ^a^	15.82 ± 1.56 ^a^	13.1 ± 1.47 ^b^
C22:5N3	15.9 ± 3.60	14.89 ± 2.29	18.92 ± 2.55
C22:5N6	3.44 ± 0.61	3.43 ± 0.54	3.5 ± 0.77
C22:6N3	4.14 ± 0.99	3.92 ± 1.24	5.73 ± 1.80
C23:0	0.17 ± 0.02 ^a^	0.19 ± 0.03 ^b^	0.16 ± 0.03 ^a^
C24:0	0.32 ± 0.03 ^a^	0.41 ± 0.08 ^b^	0.31 ± 0.04 ^a^
C24:1	0.51 ± 0.07 ^a^	0.51 ± 0.09 ^a^	0.34 ± 0.04 ^b^
C6:0	0.2 ± 0.04	0.22 ± 0.04	0.21 ± 0.02
C8:0	0.16 ± 0.03	0.18 ± 0.03	0.18 ± 0.02
EFA	332.64 ± 75.79 ^a^	311.33 ± 56.08 ^a^	474.33 ± 83.23 ^b^
SFA	510.98 ± 87.30	533.88 ± 71.18	573.38 ± 85.64
UFA	1093.24 ± 216.67	1079.29 ± 100.55	1299.09 ± 230.19
MUFA	624.17 ± 151.53	624.24 ± 65.66	679.65 ± 148.06
PUFA	469.07 ± 94.96 ^a^	455.05 ± 76.04 ^a^	619.44 ± 91.13 ^b^
ω-6 PUFA	423.92 ± 87.9 ^a^	411.74 ± 73.42 ^a^	569.58 ± 91.25 ^b^
ω-3 PUFA	25.72 ± 5.01 ^a^	24.16 ± 3.68 ^a^	33.45 ± 4.17 ^b^

Note: Different letters indicate significant differences (*p* < 0.05). SQB: Shiqi white pigeon; SQH: Shiqi grey pigeon; MMS: Mimas white pigeon.

**Table 2:** Free amino acid profiles of different pigeon groups.

Item	SQB (*n* = 6)	SQH (*n* = 6)	MMS (*n* = 6)
Alanine	4.44 ± 1.89	4.28 ± 1.11	4.00 ± 0.34
Arginine	0.15 ± 0.07	0.14 ± 0.04	0.11 ± 0.02
Asparagine	0.05 ± 0.01	0.05 ± 0.01	0.04 ± 0.01
Aspartate	12.13 ± 2.24	12.5 ± 2.45	10.39 ± 1.55
Citrulline	0.09 ± 0.02	0.15 ± 0.08	0.14 ± 0.08
Cysteine	0.01 ± 0.01	0.01 ± 0.01	0.01 ± 0.01
Cystine	8.94 ± 2.95 ^a^	9.55 ± 1.91 ^a^	12.23 ± 1.75 ^b^
Glutamate	2.07 ± 0.43	2.07 ± 0.53	1.95 ± 0.31
Glutamine	0.44 ± 0.05	0.45 ± 0.05	0.46 ± 0.05
Glycine	9.92 ± 1.60	11.62 ± 1.95	7.84 ± 2.00
Histidine	13.74 ± 4.33	12.01 ± 2.27	10.72 ± 2.04
Hydroxyproline	0.38 ± 0.28	0.33 ± 0.19	0.38 ± 0.14
Isoleucine	1.19 ± 0.11	1.18 ± 0.02	1.14 ± 0.04
Leucine	0.79 ± 0.19	0.8 ± 0.02	0.65 ± 0.12
Lysine	2.39 ± 0.89	2.33 ± 0.19	2.10 ± 0.34
Methionine	1.76 ± 0.21 ^a^	1.56 ± 0.22 ^a^	1.21 ± 0.23 ^b^
Ornithine	0.07 ± 0.01	0.08 ± 0.04	0.06 ± 0.01
Phenylalanine	2.41 ± 0.34 ^a^	2.31 ± 0.16 ^a^	1.84 ± 0.21 ^b^
Proline	2.84 ± 0.16 ^a^	3.00 ± 0.63 ^a^	3.20 ± 0.23 ^b^
Serine	11.28 ± 0.80	9.93 ± 2.18	9.75 ± 1.57
Taurine	8.30 ± 1.82	7.42 ± 0.90	10.16 ± 2.28
Threonine	0.29 ± 0.10	0.22 ± 0.06	0.41 ± 0.09
Tryptophan	0.01 ± 0.00	0.01 ± 0.00	0.01 ± 0.00
Tyrosine	2.86 ± 0.42 ^a^	2.78 ± 0.26 ^a^	2.11 ± 0.22 ^b^
Valine	2.05 ± 0.25 ^a^	1.72 ± 0.24 ^b^	1.57 ± 0.26 ^b^
SAA	28.78 ± 3.21 ^a^	29.05 ± 4.71 ^a^	25.20 ± 2.02 ^b^
DAA	42.86 ± 4.76 ^a^	43.33 ± 7.04 ^a^	36.14 ± 2.08 ^b^
BCAA	4.03 ± 0.53 ^a^	3.69 ± 0.24 ^a^	3.36 ± 0.28 ^b^
EAA	24.63 ± 4.36 ^a^	22.12 ± 2.76 ^a^	19.65 ± 2.10 ^b^
TAA	90.00 ± 9.59	87.87 ± 8.00	83.81 ± 3.32
SAA/TAA	0.32 ± 0.02	0.33 ± 0.03	0.30 ± 0.02
DAA/TAA	0.48 ± 0.04 ^a^	0.49 ± 0.05 ^a^	0.43 ± 0.02 ^b^
EAA/TAA	0.27 ± 0.03 ^a^	0.25 ± 0.02 ^a^	0.23 ± 0.02 ^b^

Note: Different letters indicate significant differences (*p* < 0.05). SQB: Shiqi white pigeon; SQH: Shiqi grey pigeon; MMS: Mimas white pigeon.

## Data Availability

The raw RNA-seq data presented in this study have been deposited in the China National GeneBank DataBase (CNGBdb) repository under the accession number CNP0008908.
